# Multigroup latent class model of musculoskeletal pain combinations in children/adolescents: identifying high-risk groups by gender and age

**DOI:** 10.1186/s10194-018-0880-0

**Published:** 2018-07-13

**Authors:** Iman Dianat, Arezou Alipour, Mohammad Asghari Jafarabadi

**Affiliations:** 1 Department of Occupational Health and Ergonomics, Tabriz University of Medical Sciences, Tabriz, Iran; 2 Road Traffic Injury Research Centre, Faculty of Health, Tabriz University of Medical Sciences, Tabriz, Iran; 3 Department of Statistics and Epidemiology, Faculty of Health, Tabriz University of Medical Sciences, Tabriz, 14711 Iran

**Keywords:** Age–specific, Gender–specific, Pain combination, Schoolchildren, Widespread pain, Multigroup LCA

## Abstract

**Background:**

To investigate the combinations of Musculoskeletal pain (MSP) (neck, shoulder, upper and low back pain) among a sample of Iranian school children.

**Methods:**

The MSP combinations was modeled by latent class analysis (LCA) to find the clusters of high–risk individuals and multigroup LCA taking into account the gender and age (≤ 13 years and ≥ 14 years of age categories).

**Results:**

The lowest and highest prevalence of MSP was 14.2% (shoulder pain in boys aged ≥14 years) and 40.4% (low back pain in boys aged ≤13 years), respectively. The likelihood of synchronized neck and low back pain (9.4–17.7%) was highest, while synchronized shoulder and upper back pain (4.5–9.4%) had the lowest probability. The probability of pain at three and four locations was significantly lower in boys aged ≥14 years than in other gender–age categories. The LCA divided the children into *minor*, *moderate*, and *major pain classes*. The likelihood of shoulder and upper back pain in the *major pain class* was higher in boys than in girls, while the likelihood of neck pain in the *moderate pain class* and low back pain in the *major pain class* were higher in children aged ≥14 years than those aged ≤13 years. Gender–age specific clustering indicated a higher likelihood of experiencing *major pain* in children aged ≤13 years.

**Conclusions:**

The findings highlight the importance of gender– and age–specific data for a more detailed understanding of the MSP combinations in children and adolescents, and identifying high-risk clusters in this regard.

## Background

Musculoskeletal pain (MSP) is an extremely common health problem in both genders and in all age groups all around the world [1, 2]. MSP is the most common cause of severe long-term pain and physical disability with significant cost to the individual and society [2, 3]. The current burden of MSP is substantial, and is predicted to increase in both the developed and developing countries [3, 4].

Recent evidence has shown that MSP is very common among school children and youth [5–7]. According to the literature, the reported incidence of neck, shoulder and spinal pain in school children and adolescents ranges from 7% to 74% [5, 6, 8–10]. There is evidence that MSP in childhood and adolescence is a contributory factor for experiencing such complaints in adulthood [11–13]. Therefore, improvement of the understanding of the characteristics of MSP among children and adolescents is necessary for designing appropriate interventions to prevent this phenomenon.

**Fig. 1:**
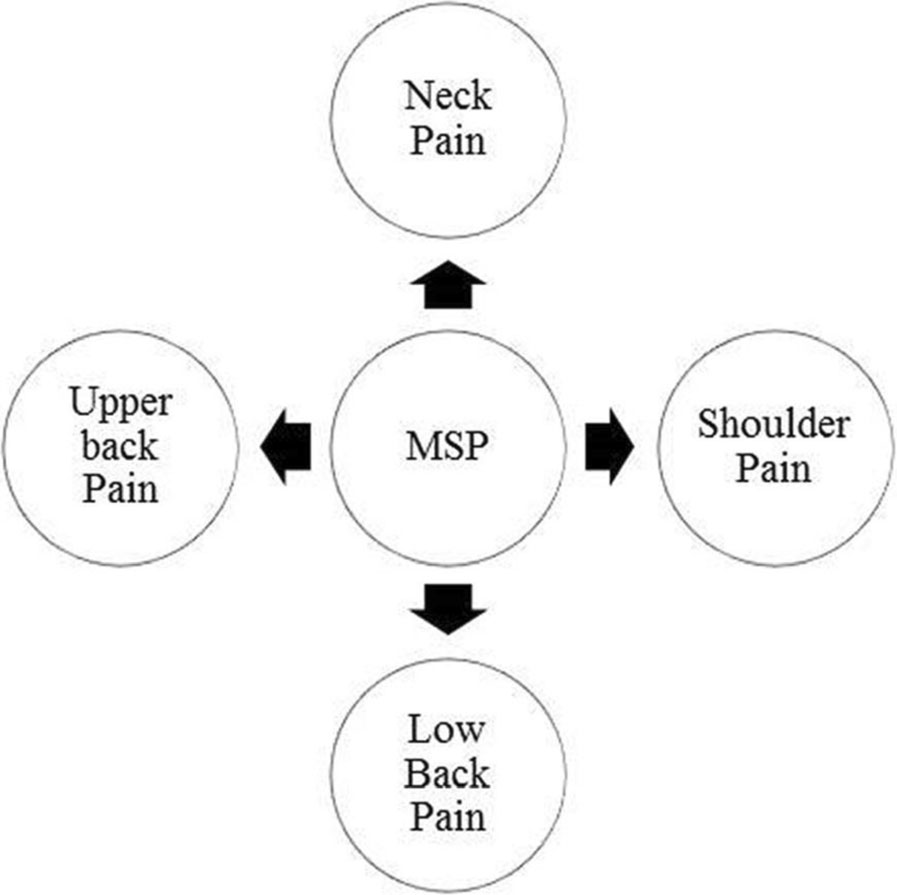
Associations of all pain locations due to an underlying latent factor

So far, most epidemiological studies on MSP in children and adolescents have evaluated one or at most two pain locations concurrently. As a result, there is limited knowledge with regard to the pain in multiple anatomic locations in this group. The need for studies on MSP combinations in children and adolescents is also well emphasized [14]. In an attempt to address this issue, the present study was conducted to evaluate the MSP combinations in neck, shoulder, upper back and low back areas. Pain combinations can be best addressed by latent class analysis (LCA). However, it has been acknowledged that LCA has been little used in the field of medical research [15], and particularly for evaluation of MSP [14]. LCA provides assessment of whether associations between observed categorical variables (e.g. neck, shoulder, upper back and low back pain in this study) can be described by the presence of an unobserved categorical variable (risk group defined by MSP) (Fig. 1). To better understand the causal mechanisms of the MSP combinations, the LCA can be utilized to determine whether the risk group defined by the MSP adequately gives details on the neck, shoulder, upper back and low back pain combinations among children and adolescents. In addition, information on gender and age subgroups can provide a basis for better identifying high–risk groups (gender– and age–specific groups) and developing interventions as well as dealing with MSP in this population. Therefore, the objective of this study was to investigate the combinations of MSP and clusters of high risk individuals among school children and adolescents using LCA and multigroup gender– and age–specific LCA.

## Methods

### Study population and procedure

This cross-sectional analytical study was performed in the city of Tabriz, Iran. Tabriz is the capital of East Azerbaijan province in North-western Iran and the fourth major city in Iran with a population of about 1.58 million. School children aged 11–15 years and were in grades 6–8 were included in the study. A three-stage sampling method was utilized to acquire a representative sample of schoolchildren. In the first stage, five educational districts were chosen as strata. In the second stage, a total of 40 schools were selected randomly from these districts, which comprised four girl’s schools and four boy’s schools. Finally, within each school, the study participants were selected randomly by simple random sampling scheme. The sample size of the study was calculated to be approximately 1105 cases, based on the main outcome of the study, the prevalence of MSP (the minimum about 7% to reach maximum sample size), considering 95% confidence, a precision about 20% and using sample size formula on single proportion in the descriptive studies. Taking into account the design effect about 1.5, the sample size increased to 1611 cases. The samples were allocated to each district and school based on a proportion to size scheme.

### Ethics approval and consent to participate

Before any participation, permission was taken from the education department of Tabriz as well as the school authorities involved. Additionally, the parental permission and informed consent for school children was also taken asking for them to contribute in the study. The study was approved by institutional review board of Tabriz university of medical sciences (code: IR.TBZMED.1394.1166). The research was conducted in accordance with the Declaration of the World Medical Association and the Helsinki Declaration on human subjects testing.

### Data collection

The data were collected using a checklist consisting of demographic and MSP data after confirmation by ethics committee. The demographic variables included gender (girls: 1, boys: 2) and age (years). The data on MSP included neck, shoulder, upper back and low back complaints during the preceding month was evaluated using a pre-shaded manikin picture showing the pain location and the modified standardized Nordic Musculoskeletal Questionnaire (NMQ) [16]. The response alternatives were: “no” and “yes”. The Farsi version of the NMQ with an established reliability and validity was used in the study [17].

### Statistical analyses

Data were analysed using LCA utilizing SAS 9.4 PROC LCA software to find the clusters of individuals by similar pattern of reported MSP [18]. The reports of symptoms were dichotomized as neck pain (no = 0, yes = 1), shoulder pain (no = 0, yes = 1), low back pain (no = 0, yes = 1), and upper back pain (no = 0, yes = 1). Simple LCA was used to estimate the probability and class membership. The study participants were assigned to the latent class clusters with the highest posterior probability as calculated based on Bayes’ theorem. A successful model fit was evaluated by a Chi-square Goodness of Fit (GOF) statistic *p* > 0.05. The conditional independence assumption was intuitively fulfilled since the measures of symptoms in anatomical sites were independently assessed. Multigroup LCA was also used to assess various features of data taking into account the effect of gender (girls = 1 and boys = 2) and age (≤ 13 years = 1 and ≥ 14 years = 2) as well as gender–age combinations (girls ≤13 years = 1, girls ≥14 years = 2, boys ≤13 years = 1, and boys ≥14 years = 4) as the grouping variables. The multigroup models were fitted based on three number of classes obtained in the first step taking into account the effect of gender, age as well as gender–age as the grouping variables in the model. To achieve an appropriate number of classes, a sequence of models were fitted with two, three and four number of classes, and the optimal model was selected by Akaike’s Information Criterion (AIC) [19] and Bayesian Information Criterion (BIC) [20]. A smaller AIC and BIC for a particular model proposes a preferable model. A 5% decrease in these measures was used to reach the conclusion. In addition, model interpretability was also considered (e.g. on the basis of the item-response probabilities, each class was distinguishable from others and no class was trivial in size) so that a meaningful label was assigned to each class. In multiple-group LCA, both the class memberships and item response probabilities can change across groups, and therefore measurement invariance can be empirically verified across groups. The parameters were estimated by the maximum likelihood procedure using the EM algorithm. The Newton-Raphson technique was used to estimate the parameters. The convergence index used was the maximum absolute deviation (MAD) to achieve the iteration limit. Missing data on the latent class and latent status indicators were permitted and treated under the assumption that data were missing at random (MAR). Any record with missing data on grouping variables specified in the model was eliminated from the analysis.

## Results

### Demographic data

A total of 1611 school children participated in the study, of which the percentage of gender–age combinations were 29.8%, 23.1%, 22.9% and 24.2% for girls ≤13 years, girls ≥14 years, boys ≤13 years and boys ≥14 years of age categories, respectively.

The mean weight of the participants were 49.7 (SD 11.36) kg; [girls ≤13 years: 48.1 (SD 10.5) kg, girls ≥14 years: 47.2 (SD 11.7) kg, boys ≤13 years: 51.1 (SD 10.1) kg and boys ≥14 years: 53.2 (SD 12.2) kg] and BMI, 19.9 (3.83) kg/m^2^ [girls ≤13 years: 19.6 (SD 3.8) kg/m^2^, girls ≥14 years: 20.0 (SD 4.2) kg/m^2^, boys ≤13 years: 19.8 (SD 3.3) kg/m^2^ and boys ≥14 years: 20.3 (SD 4.0) kg/m^2^]. More details on characteristics of the physical leisure activity, use of schoolbags and psychological trait among the schoolchildren are presented in [5, 6].

### Prevalence of symptoms

The prevalence of neck, shoulder, low back, and upper back symptoms were 27.9% (95% Confidence Interval (CI): 25.8–30.2%), 20.7% (95% CI: 18.8–22.8%), 34.3% (95% CI: 32.0–36.7%) and 19.0% (95% CI: 17.1–21.0%), respectively. Also 31.5% (95% CI: 29.3–33.9%) of participants reported pain in any of the four body regions.

### Gender–age differences in prevalence of symptoms in different body regions

Table 1 shows that prevalence of MSP in gender–age categories in different body regions. The results for *pain at one location* showed a significantly lower prevalence of shoulder symptoms in boys aged ≥14 years and higher prevalence of low back symptoms in boys and girls aged ≤13 years (*p* < 0.05) than in other gender–age categories. The results for *pain at 2 locations* showed significantly lower prevalence of neck/shoulder and shoulder/upper back symptoms in boys aged ≥14 years as well as higher prevalence of shoulder/low back and low back/upper back symptoms in boys and girls aged ≤13 years (*p* < 0.05) than in other gender–age categories. The results for *pain at 3 locations* and *pain at all 4 locations* showed significantly lower prevalence of symptoms in boys ≥14 years of age category (*p* < 0.05) than in other groups.

**Table 1:** Prevalence of MSP (*n* (%)) in gender–age categories in different body regions

	Gender–age categories				*P*-value*
	Girls ≤13 years	Girls ≥14 years	Boys ≤13 years	Boys ≥14 years	
	*n* (%)	*n* (%)	*n* (%)	*n* (%)	
Pain at 1 location					
Neck pain	136 (28.9%)	99 (27.2%)	112 (31.0%)	90 (23.6%)	0.135
Shoulder pain	108 (23.0%)^a^	77 (21.2%)^a^	87 (24.1%)^a^	**54 (14.2%)** ^**b**^	**0.003**
Upper back pain	84 (17.9%)	64 (17.6%)	83 (23.0%)	67 (17.6%)	0.164
Low back pain	**181 (38.5%)** ^**a**^	111 (30.5%)^b^	**146 (40.4%)** ^**a**^	105 (27.6%)^b^	**< 0.001**
Pain at 2 locations					
Neck/shoulder pain	190 (40.4%)^a^	145 (39.8%)^a^	155 (42.9%)^a^	**116 (30.4%)** ^**b**^	**0.002**
Shoulder/upper back pain	166 (35.3%)^a^	121 (33.2%)^a,b^	136 (37.7%)^a^	**104 (27.3%)** ^**b**^	**0.018**
Shoulder/low back pain	**222 (47.2%)** ^**a,b**^	149 (40.9%)^b,c^	**191 (52.9%)** ^**a**^	139 (36.5%)^c^	**< 0.001**
Low back/upper back pain	**171 (36.4%)** ^**a**^	101 (27.7%)^b^	**131 (36.3%)** ^**a**^	108 (28.3%)^b^	**0.006**
Neck/low back pain	161 (34.3%)	120 (33.0%)	130 (36.0%)	123 (32.3%)	0.722
Neck/upper back pain	150 (31.9%)	99 (27.2%)	101 (28.0%)	95 (24.9%)	0.145
Pain at 3 locations					
Neck/shoulder/low back pain	264 (56.2%)^a,b^	193 (53.0%)^b,c^	224 (62.0%)^a^	**178 (46.7%)** ^**c**^	**< 0.001**
Neck/shoulder/upper back pain	227 (48.3%)^a^	170 (46.7%)^a^	179 (49.6%)^a^	**147 (38.6%)** ^**b**^	**0.010**
Neck/low back/upper back pain	267 (56.8%)^a,b^	182 (50.0%)^b,c^	211 (58.4%)^a^	**180 (47.2%)** ^**c**^	**0.004**
Shoulder/low back/upper back pain	251 (53.4%)^a,b^	172 (47.3%)^b,c^	215 (59.6%)^a^	**170 (44.6%)** ^**c**^	**< 0.001**
Pain at all 4 locations					
Neck/shoulder/low back/upper back pain	286 (60.9%)^a,b^	206 (56.6%)^b,c^	236 (65.4%)^a^	**197 (51.7%)** ^**c**^	**0.001**

*P-value based on Chi-Squared test (using exact procedure)

In each row, each subscript letter denotes a subset of gender–age categories whose column proportions do not differ significantly from each other at the 0.05 level. Significant differences are shown in bold

### Choosing the number of latent classes in LCA

A smaller AIC (32.36) and BIC (107.75) for a model by three classes support this model compared to the models with two and four number of classes. Additionally, each class was distinguishable from others on the basis of item-response probabilities, so that a meaningful label was assigned to each class. The first class included those participants (60.2%) with small/moderate value (around 0.3 or lower) of probability of pain in all anatomic locations which was assigned as *moderate pain class*. The second class consisted of participants (7.1%) with high value (> 0.65) of probability of pain in all anatomic locations, which was named as *major pain class*. A small probability of pain (around 0.1 or lower) was observed in the third class with 32.7% of participants as *minor pain class* (Table 2).

**Table 2:** Membership and item response probabilities in general LCA modeling

Class name	minor pain class	moderate pain class	major pain class
Membership probabilities (%)	32.7%	60.2%	7.1%
MSP			
Neck pain	< 0.01^a^	0.36	0.88
Shoulder pain	0.04	0.24	0.70
Upper back pain	< 0.01	0.24	0.65
Low back pain	0.13	0.38	1.00

^a^Rho estimates (item response probabilities) were presented

### Gender–specific LCA modeling

The results of gender–specific LCA modeling are presented in Table 3. As can be seen from this table, the *minor pain class* consisted of 30.7% of girls and 34.5% of boys. With the same pattern of difference, the *moderate pain class* involved 57.9% and 61.6% of girls and boys, respectively. While the *major pain class* had a higher rate of pain in girls (11.4%) compared to boys (4.0%), the test of invariance across gender groups showed no significant difference between girls and boys in the pain clusters (*p* > 0.05). The findings indicated that the likelihood of shoulder and upper back pain in the *major pain class* was considerably higher in boys than girls.

**Table 3:** Membership and item response probabilities in gender-specific LCA modeling

	Gender	Latent class		
		minor pain class	moderate pain class	major pain class
Class Membership Probabilities (%)	Girl	30.7%	57.9%	**11.4%**
	Boy	34.5%	61.6%	**4.0%**
MSP				
Neck pain	Girl	< 0.01 #	0.35	**0.84**
	Boy	< 0.01	0.35	**0.94**
Shoulder pain	Girl	0.02	0.27	**0.61**
	Boy	0.04	0.21	**0.79**
Upper back pain	Girl	< 0.01	0.25	**0.50**
	Boy	< 0.01	0.23	**0.95**
Low back pain	Girl	**0.18**	0.38	1.00
	Boy	**0.08**	0.36	1.00

#: Rho estimates (item response probabilities) were presented. Considerable differences between boys and girls are shown in bold (≥ 0.1). Test the invariance across gender groups (Chi^2^_dif_ (12) = 16.76, *P*-value = 0.159)

### Age–specific LCA modeling

Table 4 shows the results of age–specific LCA modeling. Based on the test of invariance, there was significant difference across children ≤13 years and ≥ 14 years of age categories in membership probability (*p* < 0.05). The *minor pain class* involved a higher percentage of children aged ≥14 years (61.4%) compared to those aged ≤13 years (29.1%). In the *moderate pain class,* children aged ≤13 years (63.9%) were more probable to experience pain than those aged ≥14 years (33.3%). The *major pain class* consisted of 7.0% and 5.3% of children aged ≤13 years and ≥ 14 years, respectively. The results showed considerable differences in the neck and low back pain in the *moderate pain class* as well as neck pain in the *major pain class* across age categories.

**Table 4:** Membership and item response probabilities in age-specific LCA modeling

	Age (years)	Latent class		
		minor pain class	moderate pain class	major pain class
Class Membership probabilities (%)	≤ 13	29.1%	63.9%	7.0%
	≥ 14	61.4%	33.3%	5.3%
MSP				
Neck pain	≤ 13	0.07 #	**0.30**	**0.97**
	≥ 14	< 0.01	**0.71**	**0.70**
Shoulder pain	≤ 13	< 0.01	0.27	0.69
	≥ 14	0.09	0.29	0.71
Low back pain	≤ 13	0.02	0.21	**0.57**
	≥ 14	0.08	0.30	**1.00**
Upper back pain	≤ 13	**< 0.01**	0.44	1.00
	≥ 14	**0.24**	0.42	1.00

#: Rho estimates (item response probabilities) was presented. Considerable differences between age groups are shown in bold (≥0.1). Test the invariance across age groups (Chi2_dif_ (12) =22.46, *P*-value = 0.033)

### Gender–age specific LCA

The results of gender and age (combined)-specific LCA modeling are presented in Table 5. The results indicated that boys (77.8%) and girls (59.7%) aged ≥14 years were the most probable groups for experiencing MSP in the *minor* and *moderate pain classes*, respectively. Girls (30.0%) and boys (34.7%) aged ≤13 years were the most probable groups to experience MSP in the *major pain class*. However, the test of invariance across gender–age categories showed no significant difference between gender–age categories in the pain clusters (*p* > 0.05). Nevertheless, the item response probabilities showed considerable differences across gender–age categories. In the *major pain class*, girls aged ≥14 years were more likely to experience pain in all four anatomical areas than other groups. In the *moderate pain class*, the likelihood of neck and shoulder complaints in boys aged ≥14 years and also low back pain in girls aged ≥14 were higher than in other categories.

**Table 5:** Membership and item response probabilities in gender–age (combined) specific LCA modeling

	Gender-age categories	Latent class		
		minor pain class	moderate pain class	major pain class
Class Membership Probabilities (%)	Girls ≤13	38.8%	31.2%	**30.0%**
	Girls ≥14	35.5%	**59.7%**	4.8%
	Boys ≤13	28.6%	36.7%	**34.7%**
	Boys ≥14	**77.8%**	5.4%	16.8%
MSP				
Neck pain	Girls ≤13	< 0.01#	0.39	0.55
	Girls ≥14	0.10	0.32	**1.00**
	Boys ≤13	0.14	0.10	0.67
	Boys ≥14	0.13	**0.49**	0.66
Shoulder pain	Girls ≤13	< 0.01	0.28	0.48
	Girls ≥14	0.07	0.25	**0.75**
	Boys ≤13	< 0.01	0.19	0.49
	Boys ≥14	0.03	**1.00**	0.39
Low back pain	Girls ≤13	0.22	< 0.01	**1.00**
	Girls ≥14	< 0.01	**0.43**	**1.00**
	Boys ≤13	**0.63**	< 0.01	0.65
	Boys ≥14	0.22	< 0.01	0.65
Upper back pain	Girls ≤13	0.08	0.18	0.31
	Girls ≥14	< 0.01	0.23	**0.87**
	Boys ≤13	0.08	0.06	0.53
	Boys ≥14	0.09	< 0.01	0.63

#: Rho estimates (item response probabilities) was presented. Considerable differences between gender-age categories are shown in bold (≥ 0.1). Test the invariance across age groups (Chi2_dif_ (36) = 44.78, *P*-value = 0.150)

## Discussion

Although there are various studies on MSP, pain combinations in children and adolescents are poorly addressed in the literature [14]. The present study was therefore conducted to investigate the combinations of MSP among school children using LCA and multigroup gender– and age–specific LCA. One of the main findings of this study was that the MSP occurred frequently at multiple sites in the study population, which is in line with the findings of previous studies conducted in this regard [14, 21].

From a descriptive point of view, the lowest and highest levels of recorded MSP was 14.2% (shoulder pain in boys ≥14 years of age) and 40.4% (low back pain in boys ≤13 years of age), respectively. With regard to synchronized pain, the likelihood of experiencing synchronized neck and low back pain (prevalence rate was between 9.4% and 17.7%) was highest, while synchronized shoulder and upper back pain (prevalence rate was between 4.5% and 9.4%) had the lowest probability. This is similar to the findings for slightly older (16 and 18 year olds) children reported by Auvinen et al. (2009) [14]. The probability of pain at two locations was relatively the same for both genders as well as for both age categories. However, the probability of pain at three and four locations was significantly lower in boys aged ≥14 years than in other gender–age categories. This finding is relatively similar to the findings of Auvinen et al. (2009) [14], who found that MSP generally occurred more frequently in girls than in boys of the same age. This is also, in part, similar to the findings of other studies, which have reported significant associations between age and pains at multiple locations in school children and adolescents [14, 21, 22]. Also, it should be noted that the probability of pain in multiple locations had a direct relationship with the number of locations, which is also similar to the findings of Auvinen et al. (2009) [14].

It is of interest to note that this study applied multigroup LCA for better understanding and a more detailed analysis of the MSP in school children and the results showed a three-class unobserved factor explaining associations among the musculoskeletal observed components. The associations between this factor and observed musculoskeletal components were fairly modeled totally and in gender and age subgroups, which is relatively new to the literature. In summary, the LCA showed promise in expanding the current understanding of the MSP. The results of modeling demonstrated that a three-class latent factor, which is *moderate pain*, *minor pain* and *major pain*, can best describes associations among observed features of the MSP among all age categories of both genders in the studied population. Based on the results of the LCA, similar pain profiles were observed for individuals belonging to a given class. The findings demonstrated that the percentage of children with moderate, minor and high probability of experiencing pain in all anatomical locations (representing three pain classes) were 60%, 33% and 7%, respectively. This is relatively similar to the findings of previous studies conducted among school children and adolescents [14, 22]. It is of interest to note that, in the line with our results, Auvinen et al. (2009) reported that belonging to a class with high probability of pain predicted pain at all anatomic sites [14], but with a slight difference, Adamson et al. (2007) did not find any class with a single main pain site and only showed the co-occurrence of neck and back pain among their study subjects [22]. With regard to gender–specific findings, both genders had a relatively similar pattern of pain, although the likelihood of shoulder and upper back pain in the *major pain class* was higher in boys than in girls. From an age–specific point of view, a similar pattern of pain was also observed for children ≤13 years and ≥ 14 years of age categories, although the likelihood of neck pain in the *moderate pain class* and low back pain in the *major pain class* were higher in children aged ≥14 years than those aged ≤13 years. With regard to gender–age specific clustering, the results of invariance test showed a same pattern of classes for boys and girls; although the likelihood of experiencing *major pain* was considerably higher in children aged ≤13 years than in those aged ≥14 years. These findings add to the significance of gender– and age–specific data, which is new to the literature, to develop guidelines and interventions in preventing MSP in school children and adolescents, particularly for those who are at greater risk of experiencing such complaints.

The strength of the present study can be considered by the LCA which identified high-risk clusters of school children and adolescents according to their MSP profile in a large sample. The definition of the widespread pain suggested by the American College of Rheumatology [23] or the definition offered by MacFarlane et al. (1996) (e.g. The Manchester definition) for screening fibromyalgia, require experiencing pain in at least 2 contralateral body quadrants (e.g. right or left and above or below the waist) and in the axial skeleton (e.g. spine or anterior chest) [24]. Such definitions, although they may be useful clinical tools to detect and diagnose fibromyalgia and other MSP, are too specific for describing widespread pain in epidemiological studies. It seems that assessing the MSP profile clustering by LCA, and particularly in subgroups by multigroup LCA, provide a compensation for this problem.

Individuals with MSP are typically referred to physiotherapists in primary health care and physiotherapy may relieve local pain and somehow multisite pain. In MSP, the treatment aims to support change processes that can attend to pain relief and should be time-limited, but this is not the case and was a great concern and limitation for physiotherapy. The results of Whal et al. (2018) showed the relation between being the long-term consumers of physiotherapy and self-management competency and they concluded that a treatment goal for people with MSP should include development of self-management capacity [25]. The findings of current study may provide a useful framework to taking into account the diversity of the children’/adolescents’ MSP with regard to self-management capacity when referring to MSP therapy.

Furthermore, there are many comorbidities of MSP: in particular migraine [26, 27] and fibromyalgia [28]. Also, MSP may have potential interaction with other comorbidities which may affect the therapy outcome [29].

The large sample size in a general population and considering pain combinations form gender–and age–specific perspective are the major strengths of this study. Another advantage was that the data were collected by one of the authors interviewing the school children in order to decrease the likelihood of non-participation bias and to prevent observer error (e.g. as opposed to self-reporting or parental-assisted reporting). However, as in any epidemiological study, there may be possible limitation with regard to the accuracy and reliability of self-reported data on MSP as the outcome measure. However, as it has been acknowledged, this may be the only measure to understand whether and how the school children and adolescents feel any pain or discomfort, especially in large-scale populations [5, 14]. As another limitation, we performed the LCA in a cross-sectional study and have focused on gender- and age-specific MSP clusters in the population but we didn’t taking into account the risk factors; to better find the relationship between the risk factors and MSP combinations in a LCA scheme, stronger epidemiological studies (case-control and cohort designs) are recommended. Additionally, in such a large epidemiological study, the etiologies cannot be considered. However, the results may be biased by mixing benign diseases with more serious clinical conditions. As another shortcoming, the interview concerned only the preceding month. Therefore there is the risk to include both chronic pain conditions, lasting more than 6 months, and minor episodic painful situations.

## Conclusions

In conclusion, the findings of this study add to the understanding of the MSP combinations in school children and adolescents and help to identify high-risk clusters of this population according to their MSP profile. It was shown that the MSP was frequent at multiple sites among the study population. The LCA divided the studied children into three pain clusters including *moderate pain class*, *minor pain class* and *major pain class*. The LCA demonstrated that both genders had a relatively similar pattern of pain, although the likelihood of shoulder and upper back pain in the *major pain class* was higher in boys than in girls. A similar pattern of pain was also observed for the two age groups, although the likelihood of neck pain in the *moderate pain class* and low back pain in the *major pain class* were higher in children aged ≥14 years than those aged ≤13 years. The results of gender–age specific clustering indicated a same pattern of classes for boys and girls, although the likelihood of experiencing *major pain* was considerably higher in children aged ≤13 years. These findings highlight the importance of gender– and age–specific data for better understanding and a more detailed analysis of the MSP in school children and adolescents using the LCA, which can consequently lead to developing guidelines and interventions to prevent MSP in this population (particularly for those who are at greater risk of developing MSP). The findings have also implications for assessment of MSP in epidemiological studies.

## Abbreviations

AIC: Akaike’s Information Criterion
BIC: Bayesian Information Criterion
CI: Confidence Interval
GOF: Goodness of Fit
LCA: Latent class analysis
MAR: Missing at random
MSP: Musculoskeletal pain
